# Epidemiological characteristics and disease burden of childhood neuroblastoma in Asia: trends and regional differences over the past 30 years

**DOI:** 10.3389/fped.2025.1701746

**Published:** 2025-11-28

**Authors:** Zexi Li, Jing Liu, Yurui Wu

**Affiliations:** Department of Thoracic Surgery and Oncology, Capital Center for Children's Health, Capital Medical University, Capital Institute of Pediatrics, Beijing, China

**Keywords:** neuroblastoma, childhood, Asia, disease burden, epidemiological characteristics

## Abstract

**Background:**

Neuroblastoma (NB) is the most common extracranial solid tumor in children, with significant clinical heterogeneity. Despite Asia's large pediatric population, comprehensive studies on its disease burden remain limited.

**Objective:**

This study aims to evaluate trends in NB disease burden among Asian children (0–14 years) from 1990 to 2021, examining age, sex, and socioeconomic variations.

**Methods:**

Using Global Burden of Disease (GBD 2021) data, we analyzed incidence, prevalence, deaths, and disability-adjusted life years (DALYs). Statistical analysis was performed using R (v4.5.0), with stratification by Socio-Demographic Index (SDI), age, and sex. Spatial distribution and annual percentage changes were visualized using ggplot2 and heatmaps.

**Results:**

From 1990 to 2021, NB burden increased significantly across Asia. Males showed higher burden than females, with infants (1–11 months) at greatest risk. East Asia experienced the fastest growth, while South Asia had the highest absolute burden. High-SDI regions demonstrated declining trends of disease burden but maintained the highest case numbers. Country-level variations were substantial, with Qatar and Afghanistan showing the largest increases and Kyrgyzstan the greatest reductions. SDI correlated positively with NB burden, suggesting improved detection in developed regions. Females exhibited bimodal incidence peaks (6–11 months and 5–9 years), while males peaked at 1–5 months and 2–4 years.

**Conclusion:**

The burden of NB in Asian children is characterized by age and gender differences and socioeconomic drivers. It is necessary to optimize screening and resource allocation strategies for high-risk groups.

## Introduction

Neuroblastoma (NB), accounting for 8%–10% of childhood malignancies (1), is the most common extracranial solid tumor in children, predominantly affecting those under 5 years, with peak incidence in infants (2). Originating from neural crest cells (3), NB shows substantial heterogeneity in anatomical sites, most often adrenal glands, but also abdomen, thorax, or neck (4), and in its clinical course. The International Neuroblastoma Risk Group (INRG) classification categorizes NB from low-risk tumors capable of spontaneous regression to high-risk forms with aggressive metastasis (5). Despite advances in surgery, chemotherapy, radiotherapy, immunotherapy, and targeted therapy (6, 7), high-risk NB still has <50% five-year survival and remains a leading cause of pediatric cancer mortality (8), particularly in resource-limited regions with delayed diagnosis and inadequate treatment, highlighting the need for focused epidemiological and intervention studies.

Compared with the global average, Asia bears a disproportionately high burden of childhood NB (9). While comprehensive NB epidemiological data from Western registries (e.g., SEER) have established baseline incidence and survival patterns (10), significant knowledge gaps persisted in Asian populations due to region-specific genetic susceptibilities, environmental exposures, and heterogeneous healthcare access across economically diverse nations. The current fragmentation of Asian childhood cancer registries, with varying levels of completeness, hindered cross-regional comparisons, potentially masking unique epidemiological features distinct from Western patterns (11). The standardized methodology of the Global Burden of Disease (GBD) study offered a crucial solution by integrating comparable metrics [incidence, deaths, disability-adjusted life years (DALYs)] across regions, enabling systematic investigation of diagnostic delays, treatment disparities, and population-specific risk factors that may reshape our understanding of NB's Asian disease burden (9).

Herein, this study leveraged the GBD database to conduct the first comprehensive assessment of NB burden among Asian children over the period 1990–2021, a timeframe chosen because reliable and consistent datasets have been available since 1990, enabling robust long-term trend analysis, and 2021 represents the most recent year with validated data. We analyzed incidence, deaths, and DALYs across key demographic, geoeconomic strata, and national income levels. Moreover, this research aims to establish benchmark epidemiological profiles for Asian NB, identify high-burden populations and modifiable drivers of diagnostic delays, and inform targeted interventions such as optimized screening algorithms and resource allocation strategies. The findings will address critical gaps in pediatric oncology data, providing actionable evidence for regional policy-making and equitable healthcare delivery across Asia.

## Methods

### Data sources

Utilizing the GBD 2021 (https://vizhub.healthdata.org/gbd-results/) database, which synthesized data from diverse sources including disease registries, hospital records, vital statistics, and population-based studies, this study systematically analyzed the burden of NB among Asian children aged 0–14 years from 1990 to 2021. We examined key epidemiological indicators - incidence, prevalence, mortality, and DALYs - to provide the first comprehensive assessment of temporal trends and spatial distribution across Asian subregions. Moreover, we also incorporated country-level Socio-Demographic Index (SDI) data to enable a stratified examination of temporal trends and spatial patterns across Asian subregions.

### Data analysis

All statistical analyses and visualizations were performed using R (version 4.5.0). Temporal trends in NB burden across Asia were analyzed using “ggplot2”, with stratification by SDI (high: SDI ≥ 0.81, high-middle: 0.71 ≤ SDI < 0.81, middle: 0.62 ≤ SDI < 0.71, low-middle: 0.47 ≤ SDI < 0.62, low: SDI < 0.47), age groups (0–6 days, 7–27 days, 1–5 months, 6–11 months, 12–23 months, 2–4 years, 5–9 years, and 10–14 years), and sex (male and female). Relative changes in case numbers and age-standardized rates (2021 vs. 1990) were computed and visualized. Geospatial mapping was conducted using “maps” and “ggplot2” to generate choropleth maps of disease burden and heatmaps of estimated annual percentage change (EAPC). Additionally, “ggrepel” was employed to plot correlations between SDI and age-standardized incidence, prevalence, deaths, and DALYs.

## Results

### The disease burden of childhood NB in different parts of Asia

From 1990 to 2021, Asia experienced a significant rise in NB burden across all key epidemiological indicators. The overall prevalence increased from 0.476 to 0.760 per 100,000, while incidence rose from 0.069 to 0.132 per 100,100, deaths from 0.034 to 0.061 per 100,000, and DALYs from 2.304 to 3.592 per 100,000. Sex-stratified analysis revealed consistently higher rates in males: prevalence (0.504–0.832 per 100,000), incidence (0.074–0.152 per 100,000), deaths (0.037–0.072 per 100,000), and DALYs (2.454–4.014 per 100,000). Females exhibited comparatively lower but still substantial increases: prevalence (0.445–0.681 per 100,000), incidence (0.063–0.113 per 100,000), mortality (0.031–0.052 per 100,000), and DALYs (2.145–3.157 per 100,000).

**Figure 1:**
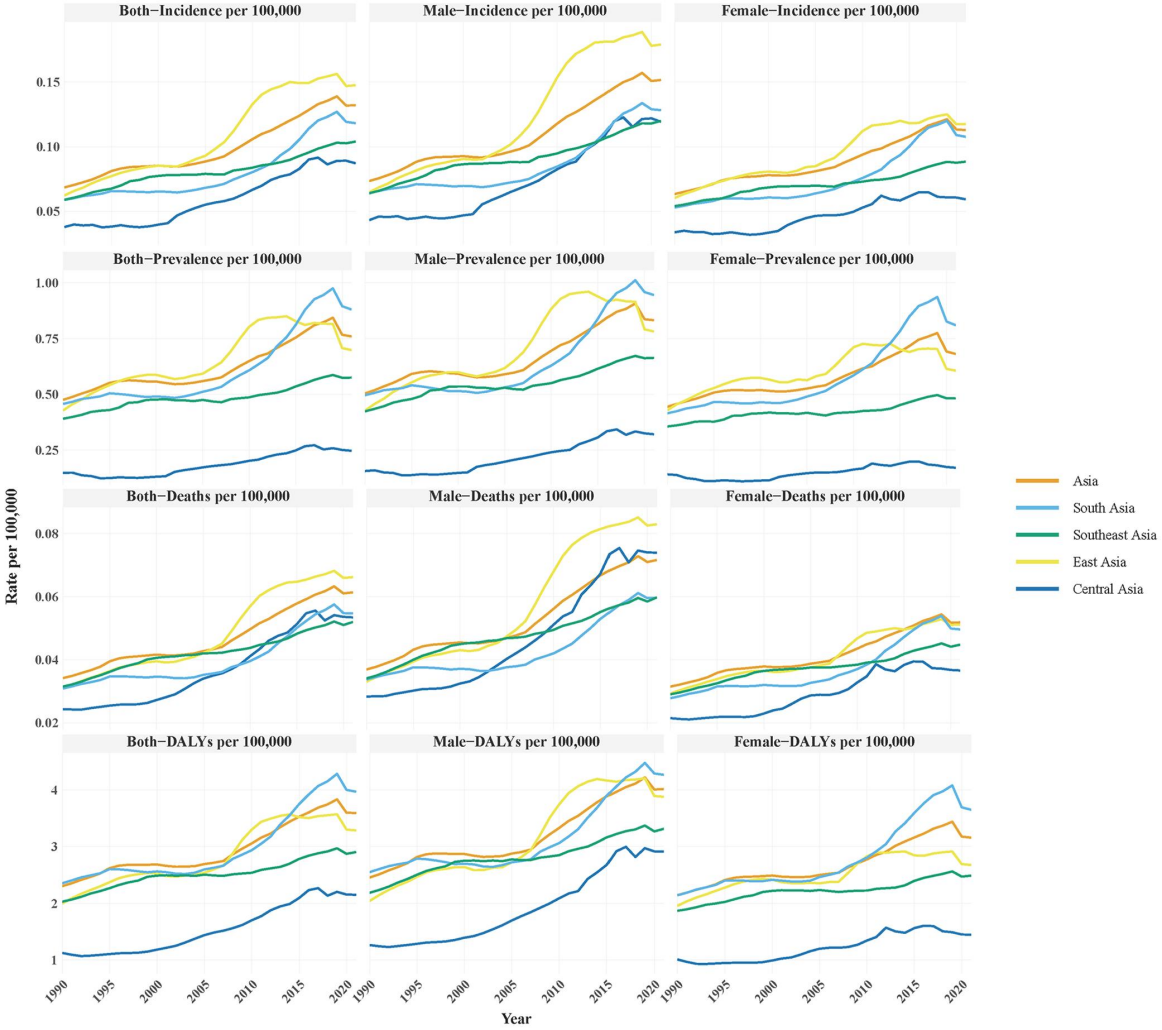
Disease burden of neuroblastoma (NB) in Asian regions (1990–2021),. focusing on incidence, prevalence, deaths, and disability-adjusted life years (DALYs) per 100,000 population for both sexes, males, and females.

Regional disparities were evident, East Asia showed the most sharply rising in incidence (0.062–0.148 per 100,000) and deaths (0.031–0.066 per 100,000), South Asia have the highest burden in prevalence (0.458 to 0.880 per 100,000) and DALYs (2.354–3.967 per 100,000). Despite exhibiting an upward trajectory, all NB burden indicators in Central Asia remain consistently lower than those observed in other Asian regions (incidence:0.038–0.087 per 100,000, prevalence: 0.149–0.247 per 100,000, deaths: 0.024–0.053 per 100,000, DALYs: 1.125–2.151 per 100,000, Figure 1).

### Trends in SDI stratification of childhood NB disease burden in Asia

**Figure 2:**
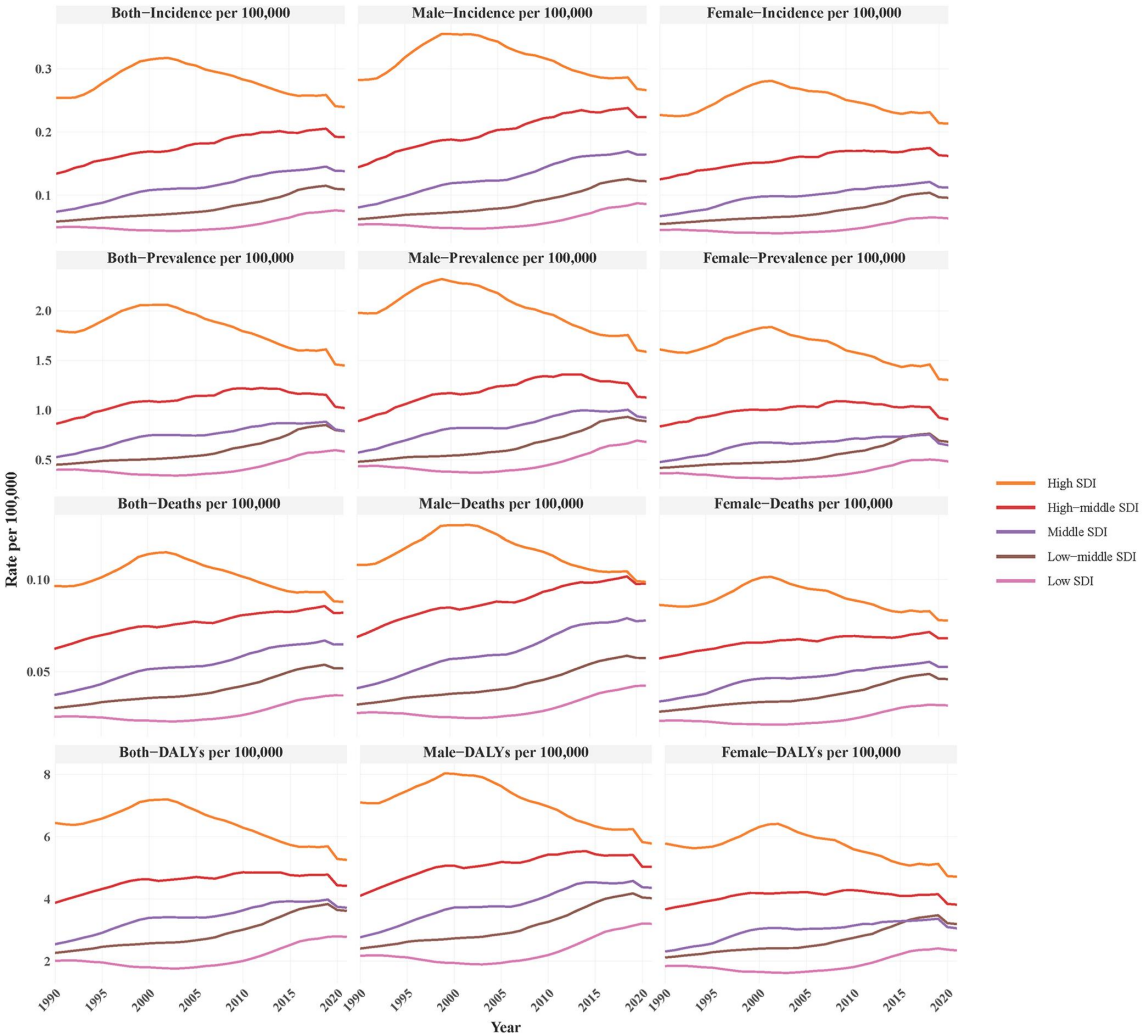
Disease burden of NB in different socio-demographic Index (SDI) groups, focusing on incidence, prevalence, deaths, and DALYs per 100,000 population for both sexes, males, and females.

The disease burden of childhood NB in Asia demonstrated pronounced SDI stratification during 1990–2021. Notably, while the high-SDI region was the only stratum exhibiting declines across all indicators (prevalence decreased from 0.254 to 0.239 per 100,000, incidence from 1.800 to 1.449 per 100,000, deaths from 0.097 to 0.088 per 100,000, and DALY rate from 6.442 to 5.253 per 100,000), its disease burden remained substantially higher than that of other SDI regions. In contrast, other SDI regions exhibited rising incidence rates, but their overall disease burden remained lower than that of high-SDI areas. This pattern was consistent across prevalence, deaths, and DALY. Notably, males consistently bore a higher disease burden than females across all SDI strata. These findings suggested that although high-SDI regions demonstrate declining trends, their absolute burden remains elevated, whereas low- and middle-SDI regions face rapidly increasing neuroblastoma rates (Figure 2).

### Age-stratified burden of NB in Asian children

**Figure 3:**
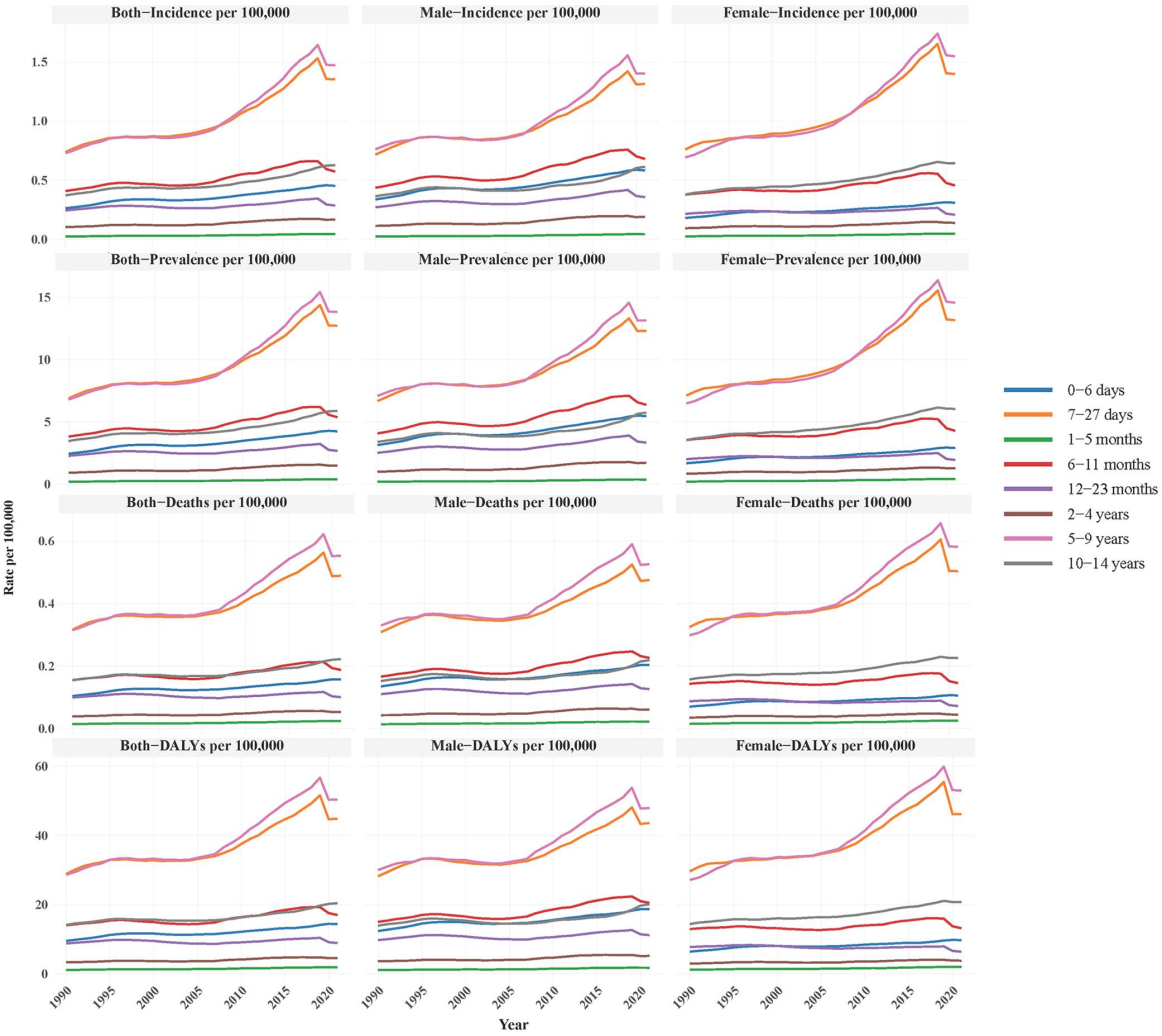
Disease burden of NB in different age groups, focusing on incidence, prevalence, deaths, and DALYs per 100,000 population for both sexes, males, and females.

From 1990 to 2021, NB disease burden among Asian children displayed pronounced age-related disparities, with infancy (1–11 months) emerging as the most critical period. This group exhibited the highest incidence, prevalence, deaths, and DALY rates, surpassing all other age groups. Infants aged 1–5 months saw incidence rise from 0.738 to 1.354 per 100,000, while those aged 6–11 months experienced a near-doubling in prevalence (6.806–13.817 per 100,000) and a sharp DALY rate increase (28.622–50.344 per 100,000). Females consistently carried a higher burden than males, with a neonatal incidence (0–6 days: 0.583 vs. 0.309 per 100,000) nearly 1.9 times higher and a 50% greater prevalence at 12–23 months (6.375 vs. 4.290 per 100,000). Notably, males aged 2–4 years were the only subgroup with declining incidence (0.214–0.207 per 100,000), while females in the same age range showed sustained increases (0.270–0.357 per 100,000), suggesting potential biological or diagnostic/therapeutic influences on sex-based disparities. Furthermore, children over 5 years maintained lower overall burdens, yet deaths rates doubled in the 10–14 age group (0.014–0.023 per 100,000), signaling emerging risks in older cohorts (Figure 3).

### Changes and EAPC in the burden of childhood NB in Asia across countries

**Figure 4:**
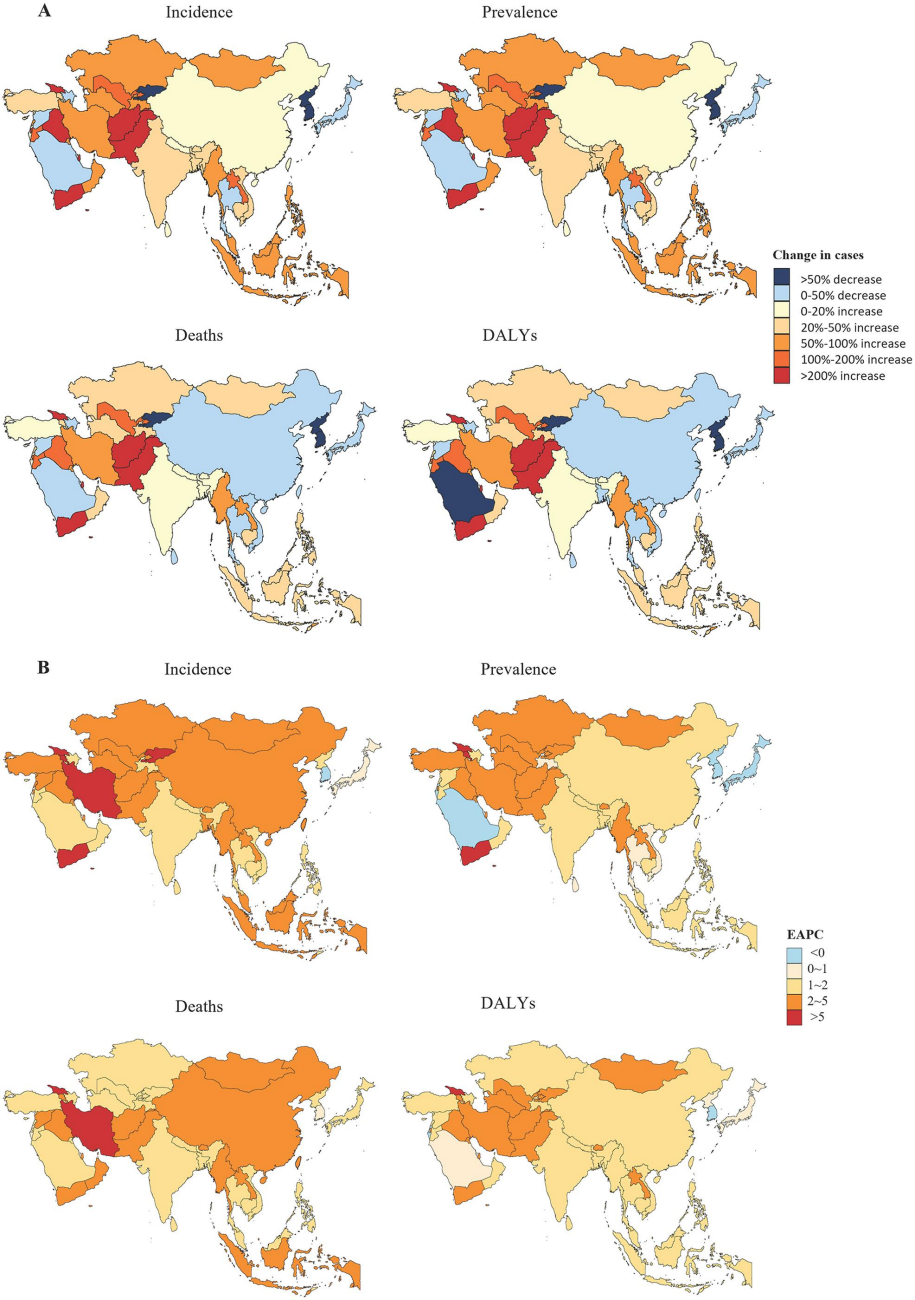
The changes of disease burden of NB in Asia. **(A)** The percentages of changes in the number of prevalence, incidence, deaths and DALYs. **(B)** The changes in the Estimated Annual Percentage Change (EAPC) of prevalence, incidence, deaths and DALYs.

The study revealed substantial variations in childhood NB burden across Asian countries between 1990 and 2021. Qatar recorded the most dramatic increases, with a 659% rise in both incident cases and prevalent cases. Afghanistan showed the highest surge in mortality-related indicators, with a 653% increase in deaths and a 647% rise in DALYs. In contrast, Kyrgyzstan achieved the most significant reductions, with 72%–75% declines across all indicators (incidence: 72%, prevalence: 72%, deaths: 74%, and DALYs: 75%). Georgia exhibited consistent increases in incidence, prevalence, deaths, and DALYs. South Korea and Singapore demonstrated steady declines, with EAPCs for incidence, prevalence, and deaths. Rapid increases were observed in Middle Eastern and Central Asian countries (e.g., Qatar, Afghanistan), while notable decreases occurred in East Asia (e.g., South Korea, Singapore) and other developed nations (Figure 4).

### Relationship between SDI and NB burden

**Figure 5:**
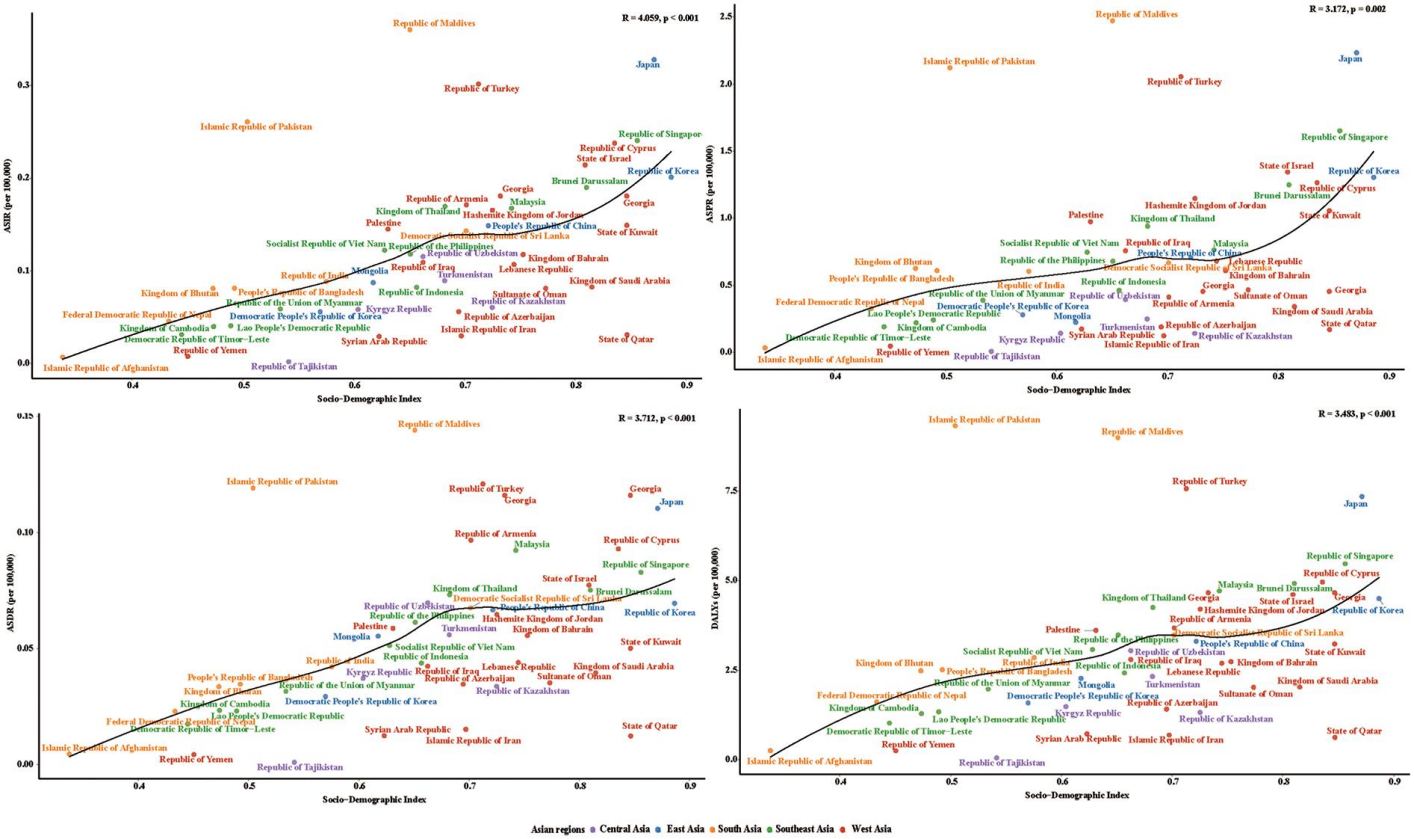
The relationship between ASIR, ASPR, ASDR, DALYs and SDI in Asian countries.

The study found a significant positive correlation between the SDI and NB burden across 48 Asian countries in 2021, with SDI showing strong associations with incidence (R = 4.059, *p* < 0.001), prevalence (R = 3.172, *p* = 0.002), deaths (R = 3.712, *p* < 0.001), and DALYs (R = 3.483, *p* < 0.001). This suggested that higher SDI levels were linked to increased case detection (likely due to improved diagnostic capacity) as well as elevated deaths and disease burden. Geographically, neuroblastoma burden remained highest in East and West Asia but showed a gradual convergence with lower-burden regions (South and Southeast Asia), indicating narrowing epidemiological disparities across the continent (Figure 5).

### NB burden of Asian children in different sex and age

**Figure 6:**
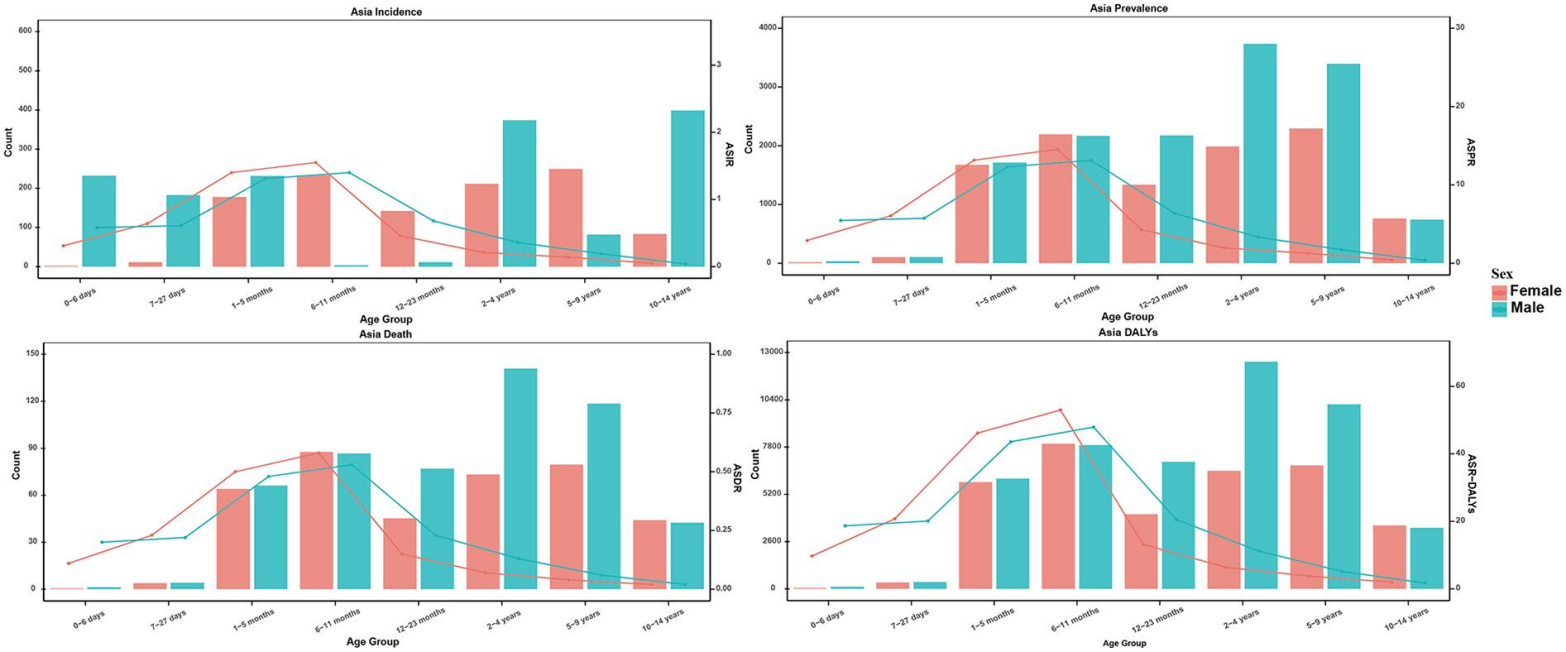
NB burden of Asian children in different sex and age, focusing on incidence, prevalence, deaths, and DALYs.

The disease burden of NB in Asian children was significantly different in sex and age distribution. The numbers of incidence, prevalence, deaths and DALYs of females showed a bimodal distribution, with incidence at 6–11 months and 5–9 years of age, respectively, while in males, the incidence peaked at 1–5 months and 2–4 years of age, with deaths and DALYs peaking at 6–11 months and 2–4 years of age. Meanwhile, the rates of incidence, prevalence, deaths and DALYs of females showed a unimodal distribution, which concentrated in 6–11 months of age. Males also showed a unimodal distribution in the above indicators, concentrated between 6 and 11 months of age. In general, the risk of prevalence and deaths of this disease was significant in infants and young children, and there were differences in the peak age and distribution characteristics between males and females, which provided an important reference for early screening and intervention of NB (Figure 6).

## Discussion

This global epidemiological study revealed distinct patterns in childhood NB burden, with Asia demonstrating a persistent incidence increase. Our analysis identified critical epidemiological features: (1) a pronounced disease peak among infants aged 6–11 months, (2) consistent male predominance across regions, and (3) significant sociodemographic disparities—high-SDI regions may showed elevated detection rates with improved survival, whereas low-SDI areas suffered from substantial underdiagnosis and treatment-access limitations that mask the true disease burden. These findings underscored the urgent need for (1) establishment of Asian-specific population registries, (2) development of developmental-stage-targeted screening protocols, and (3) strategic healthcare resource redistribution to address existing prevention and treatment disparities.

The current study demonstrated a persistent rise in childhood NB burden across Asia, driven by multiple region-specific etiological factors. First, environmental pollutants (particularly PM2.5 (12) and heavy metals (13) associated with rapid urbanization may promote tumorigenesis through epigenetic mechanisms (14). Second, population-specific genetic variants (including ALKBH3 and LIN28B polymorphisms) appear to influence disease susceptibility (15, 16). Third, the prevalent overuse of antibiotics may impair immune surveillance by inducing pediatric gut microbiota dysbiosis (17). These factors highlighted the critical need for tailored prevention strategies that address Asia's distinct environmental, genetic, and iatrogenic risk profile.

The observed sex differences in NB suggested a complex, multifactorial etiology. The higher incidence in males (18) may stem from loss of chromosome Y (19), whereas the unique bimodal age distribution in females could reflect dynamic changes in estrogen receptor signaling during development (20). Notably, the increased incidence in females and decreased incidence in males in the 2–4-year age group may indicate sex-specific variations in immune maturation timing (21). Molecular studies further support these findings, for example, MYCN-regulated microRNAs repress estrogen receptor-alpha (ESR1) expression and neuronal differentiation in human neuroblastoma (22). X-linked miRNAs may exert gender-specific tumor-suppressive effects. Sex hormones can modulate therapeutic responses by influencing immune cell infiltration in the tumor microenvironment (23). These insights not only guide translational research, such as the development of sex-specific risk prediction models, but also highlight the importance of incorporating sex-based considerations into clinical practice to optimize personalized treatment strategies.

The socioeconomic disparities in childhood NB burden exemplified the “Matthew effect” prevalent in global health (24). High SDI regions, benefiting from superior healthcare infrastructure, demonstrated an advanced epidemiological profile characterized by early detection capabilities, resulting in apparent “high incidence but low deaths” patterns (9). Conversely, low-SDI regions remain trapped in a detrimental cycle of underdiagnosis and elevated deaths rates (25). This dichotomy fundamentally mirrored structural inequities in global health resource allocation. Notably, the true disease burden in low-to-middle SDI regions may be substantially underestimated due to inadequate tumor surveillance systems and limited pathological diagnostic capacity.

The pronounced disease burden observed during infancy, particularly between 6 and 11 months of age, offered critical insights into NB pathogenesis. This high-risk window coincides with three fundamental biological transitions: (1) the decline of maternal antibodies coupled with autoimmune system maturation (26); (2) accelerated sympathetic nervous system development accompanied by dynamic nerve growth factor (NGF) and receptor expression changes (27); and (3) stabilization of gut microbiota colonization, potentially influencing the tumor microenvironment through microbiota-immune-neural crosstalk (28, 29).

Based on these findings, we speculated that embryonic molecular aberrations (e.g., neural crest cell differentiation defects) require passage through this critical period to manifest as clinically detectable tumors. This reasoning not only explains the disease's temporal pattern but also suggests a paradigm shift in prevention strategies—from static tumor detection to dynamic monitoring of developmental milestones. Future research should prioritize: (1) high-resolution molecular profiling of this critical window, (2) mechanistic studies of neurodevelopmental-immune system interactions, (3) biomarker development for early risk stratification, and (4) intervention strategies targeting developmental vulnerabilities. These investigations could transform NB management by enabling developmentally timed prevention, moving beyond conventional diagnostic approaches.

While this study characterized the disease burden of NB in Asian children, key limitations include: (1) potential underreporting due to incomplete cancer registries in low-resource regions, particularly rural areas; (2) methodological constraints where conventional age-standardization may inadequately reflect infant-specific biology and regional diagnostic variations; (3) lack of critical etiological data (environmental exposures, molecular markers like MYCN amplification) hindering mechanistic insights; and (4) retrospective design limitations affecting causal inference and survival estimates, compounded by unadjusted sociocultural influences on healthcare-seeking behaviors. These gaps underscore the need for future multicenter collaborations with improved surveillance systems, prospective designs incorporating multi-omics approaches, and culturally sensitive methodologies to advance accurate burden assessment and risk stratification.

## Conclusion

This study provides the first comprehensive epidemiological analysis of pediatric NB in Asia (1990–2021), revealing a rising disease burden with distinct demographic disparities—most notably a peak incidence in infants aged 6–11 months and significant sex-based variations. We identified stark healthcare inequalities: high-SDI regions achieved “high detection, low mortality” due to advanced medical infrastructure, while low-SDI regions faced substantial underdiagnosis, obscuring the true burden. To address these gaps, we advocate for a standardized Asian NB registry, infant-specific early diagnostic protocols, and optimized resource allocation, measures critical to achieving equitable, precision-based NB control and improved survival across socioeconomic strata.

## Data Availability

The original contributions presented in the study are included in the article/Supplementary Material, further inquiries can be directed to the corresponding author.
